# *Shigella dysenteriae* infection activates proinflammatory response through β-catenin/NF-κB signaling pathway

**DOI:** 10.1371/journal.pone.0174943

**Published:** 2017-04-21

**Authors:** Ashidha Gopal, Iyer Soumya Chidambaram, Niranjali Devaraj, Halagowder Devaraj

**Affiliations:** 1 Unit of Biochemistry, Department of Zoology, University of Madras, Chennai, Tamilnadu, India; 2 Department of Biochemistry, University of Madras, Chennai, Tamilnadu, India; University of Nebraska Medical Center, UNITED STATES

## Abstract

*Shigella dysenteriae* (*S*.*dysenteriae)* the causative agent of bacillary dysentery invades the human colonic epithelium resulting in severe intestinal inflammatory response and epithelial destruction. However, the mechanism by which *S*.*dysenteriae* infection regulates proinflammatory cytokines during intestinal inflammation is still obscure. In this study, we evaluated whether the interaction of β-catenin and NF-κB regulates proinflammatory cytokines TNF-α and IL-8 by modulating GSK-3β activity during *S*.*dysenteriae* infection in rat ileal loop model. Here we demonstrated that *S*.*dysenteriae* infection stimulate β-catenin degradation which in turn decreased the association between NF-κB and β-catenin. Also, we showed that *S*.*dysenteriae* infection increased GSK-3β kinase activity which in turn phosphorylates β-catenin for its degradation by ubiquitination and upregulates IL-8 through NF-κB activation thereby leading to inflammation. Thus these findings revealed the role of β-catenin/ NF-κB and GSK-3β in modulating the inflammatory response during bacterial infection and also showed that β-catenin acts as a critical regulator of inflammation.

## Introduction

*Shigella* species cause bacillary dysentery in humans by invasion, intracellular multiplication, spread to adjacent cells and induction of inflammatory responses in the intestinal epithelium [1]. Cytokines and chemokines are well recognized as key mediators of the inflammatory cascade causing Inflammatory Bowel Disease [2]. Among the chemokines IL-8 is best studied in several cell types including monocytes and macrophages, fibroblasts, endothelial cells and keratinocytes [3–6]. Previous reports state that human intestinal and cervical epithelial cells secrete IL-8 in response to bacterial entry and suggest that IL-8 secreted by epithelial cells may be the initial signal for the acute inflammatory response of mucosal surfaces following bacterial invasion. [7]. However, the mechanism that regulates IL-8 during bacterial invasion into epithelial cells remains unclear.

The Wnt signaling pathway has been shown to play a major role in intestinal morphogenesis and cell fate determination and in renewal of the intestinal epithelium [8–10]. Wnts are known to activate the β-catenin pathway that plays a major role in intestinal inflammation. Several reports have implied the involvement of Wnt-Frizzled signaling in the activation of proinflammatory mediators in inflammatory disorders and expression of wnts were significantly higher in patients with inflammatory bowel disease (IBD) than in non-IBD patients [11, 12]. In the absence of Wnt signals, free cytosolic β-catenin undergoes degradation via β-catenin destruction complex that contains adenomatous polyposis coli (APC), Axin that act as protein scaffolds as well as casein kinase I and glycogen synthase kinase 3β. Wnts are known to activate the β-catenin pathway, a regulator of intestinal epithelial proliferation and inflammation [13–18]. 19 different Wnt ligands have been described in mammals. In the presence of wnt ligands, β-catenin destruction complex becomes inactive and the β-catenin translocate to the nucleus where it binds LEF/TCF transcription factors and drives the transcription of wnt target genes. Reports state enteric pathogens like *Salmonella typhimurium* cause acute intestinal inflammation by activating the NF-κB pathway, which requires the ubiquitination and degradation of the inhibitory molecule IκBα [19]. NF-κB has been studied extensively in inflammation and it is known to regulate pro-inflammatory cytokines such as IL-1β, IL-2, TNF-α and chemokines such as IL-8 and reports state that β-catenin interacted with NF-κB in human colon and breast cancer cells and it was found that β-catenin could physically complex with NF-κB, resulting in a reduction of NF-κB DNA binding, transactivation activity and target gene expression [20–23]. Previous reports also state that *S*. *typhimurium* PhoP^c^ inhibits activation of the proinflammatory transcription factor NF-κB and regulates the β-catenin pathway in human epithelial cells. However, the role of NF-κB and β-catenin signaling in the regulation of proinflammatory cytokine has not yet been elucidated during *Shigella* infection in *in vivo* model.

Therefore, the present study aims to investigate the potential role of β-catenin and NF-κB signaling in regulating IL-8 that acts as a mediator of mucosal inflammation in the rat ligated intestinal loop model of *S*.*dysenteriae* infection. The results of this study suggest that stabilizing of β-catenin has a significant anti-inflammatory effect by reducing NF-κB mediated pro-inflammatory activity, thus controlling intestinal inflammation.

## Materials and methods

### Bacterial strain and growth conditions

Clinical isolates of *S*.*dysenteriae* were obtained from Department of Medical Microbiology, Christian Medical College (CMC), Vellore, India. The strains were routinely grown in Luria- Bertani (LB) broth (Himedia, Mumbai, India) at 37°C overnight.

### Shigella-Salmonella agar and virulence assay

The specificity of *S*.*dysenteriae* was assessed by Shigella-Salmonella (SS) agar. Bacterial strains were grown in Shigella-Salmonella agar at 37°C for 18h.

The virulence nature of *S*.*dysenteriae* was assessed by Congo-red dye binding assay and the procedure was followed as described previously [24]. Briefly, bacterial strains were grown in Congo red (0.01%) supplemented Tryptic soy broth containing 0.6% yeast extract and 1.5% agar at 37°C for 18h.

### Rat ileal loop infection with *S*.*dysenteriae*

Wistar strain male albino rats weighing 120-150g were obtained from TANUVAS, Madhavaram, Chennai. The protocol was approved by the Institutional Animals Ethics Committee (IAEC) of University of Madras, INDIA, (approval no IAEC No.011/02/2011). This study was carried out in accordance with the guidelines of the Committee for the purpose of Control and Supervision on Experiments on Animals (CPCSEA) and also as per S1 Checklist. Briefly, Male Wistar albino rats were fasted for 24hr prior to experimentation and the animals were anaesthetized with Ketamine/Xylazine (90/10mg/kg body weight). After making a small incision in the abdominal region, inocula of 10^9^ CFU in 0.5ml of PBS (pH 7.4) was injected into ligated ileal loops and the animals were allowed to live and sacrificed at 8hr. The loops with PBS served as control and it is mentioned as untreated. The infected loops were used for standard histological staining, immunohistochemistry, western blot and real-time PCR analysis.

### Histological examination

Immediately after sacrifice, the small intestine was removed and flushed with ice-cold phosphate-buffered saline (PBS) and divided into a number of segments to allow easy handling. The portions were slit open longitudinally and the contents were carefully removed. Next, each segment is rolled up longitudinally, with the mucosa outwards, using a wooden stick. Each of the resulting “Swiss rolls” was then carefully placed in 10% buffered formalin for paraffin-wax embedding [25]. They were cut into 4 μm sections. Paraffin embedded sections were then stained with hematoxylin and eosin.

### Immunohistochemistry

Paraffin-embedded infected ileal sections were deparaffinized in xylene and dehydrated through graded concentrations of isopropyl alcohol. After blocking the endogenous peroxidase activity with 3% H_2_O_2_, the sections were heated in 10 mM sodium citrate buffer at pH 6.0 in a microwave oven for 20 min. The slides were allowed to cool at room temperature and non-specific binding was blocked with 3% BSA for 1 hr at room temperature. The sections were incubated with primary antibodies NF-κB (dilution 1:250; Chemicon) and β-catenin (dilution 1:200; Santa Cruz, USA) overnight at 4°C in a humidified chamber. Bound antibody was detected by a horseradish peroxidase-conjugated secondary antibody. The peroxidase reaction was developed in PBS with hydrogen peroxide as substrate and diaminobenzidine (DAB) as a chromogen. Sections were counterstained with Mayer’s hematoxylin, rehydrated and mounted with DPX and then visualized under Axioskope 2d microscope, Carl Zeiss, Germany.

### Western blot

Protein lysates were prepared and protein concentrations were determined by Lowry et al [26]. Protein extracts (40 μg) were analyzed by 10% SDS-PAGE and transferred onto a nitrocellulose membrane. The membranes were blocked with 10% skimmed milk powder in TBS-T buffer (20 mM Tris–HCl, pH 7.6, 137 mM NaCl and 0.1% Tween-20) for 1hr and then incubated overnight at 4°C with primary antibody diluted in 3% skimmed milk powder in TBS-T. The following antibodies were used: p-β-catenin (Dilution 1:2000), β - catenin (Dilution 1:5000), p-GSK-3β (Y216) (Dilution 1:5000), p-GSK-3β (S9) (Dilution 1:2000), GSK-3β (Dilution 1: 2000), p-IκBα (Dilution 1:5000), NF-κB (Dilution 1:5000), IL-8 (Dilution 1:5000), TNF-α (Dilution 1:500) and Tubulin (Dilution 1:10000). The membrane was then washed thrice for 5min each with TBS-T and then incubated with corresponding secondary antibodies conjugated with HRP (Santa Cruz Biotech, USA) in TBS-T. The membrane was washed thrice for 5min each with TBS-T and then equal volume of Luminol A and B (ECL) solutions were added to the membrane. The membrane was exposed to hyperfilm and signals were detected on hyperfilm by using the enhanced chemiluminescent reagent kit (No. RPN2135- Amersham, ECL advance, Western blotting Detection Kit-UK) as per manufacturer’s protocol. Tubulin was used as an internal control.

### Co-immunoprecipitation

For immunoprecipitation analysis, the protein lysates were immunoprecipitated with β - catenin and GSK-3β with gentle rocking at 4°C overnight and pulled down with protein A agarose beads as described by Udhayakumar et al [27]. The beads were washed extensively and the bound proteins were resolved by SDS-PAGE and analyzed by immunoblotting using NF-κB and β-catenin.

### RNA extraction and real-time PCR

Total RNA was extracted using TRIZOL reagent (Bangalore Genei Pvt., Ltd., India). Reverse transcription was performed with 3μg of total RNA. The following primers were used: TNF-α, 5’-ACTGAACTTCGGGGTGATCGGTCC; reverse, 5’-GTGGGTGAGGAGCACGTA GTCG; IL-8, 5’-ACGCTGGCTTCTGACAACACTAGT; reverse, 5’-CTTCTCTGTCCTGAGACGAGAAGG; GAPDH, 5’-AGCCATGTACGTAGCCATCC; reverse 5’–CTCTCAGCTGTGGTGGTGAA; IL-8 and TNF-α was amplified with SYBR Green (BioRad) as described by Ashidha et al [28]. Each experiment was carried out in triplicate at least twice; the results are expressed as means ± SD of representative triplicates.

### Statistical analysis

Data are shown as the mean ± SD. Statistical evaluation was done by unpaired Student’s *t* test, and *p* < 0.05 was taken as a significant difference.

## Results

### *Shigella- Salmonella* agar and virulence nature of *S*.*dysenteriae*

**Fig 1:**
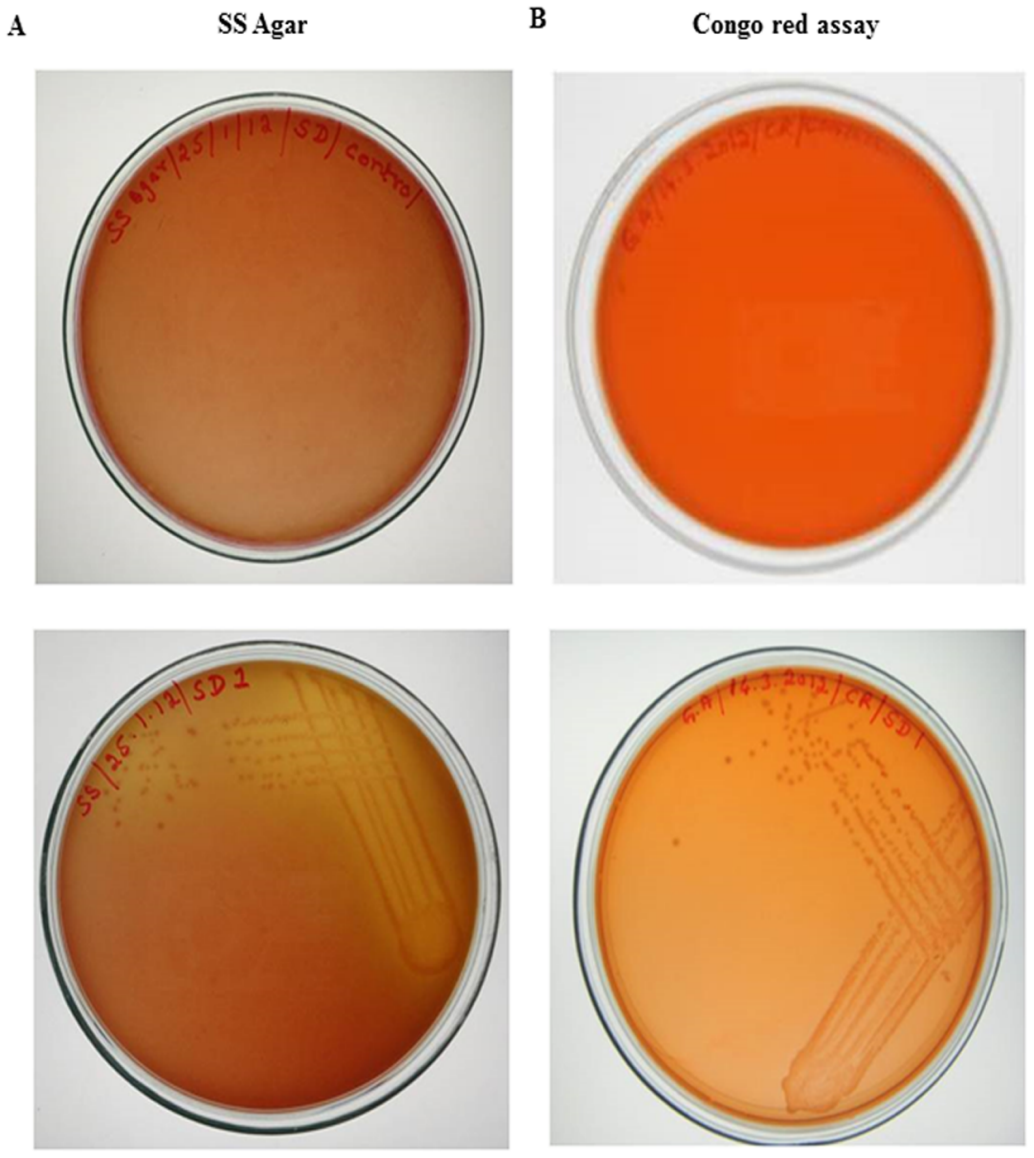
*Shigella-Salmonella* agar and Congo-red binding assay. A) *Shigella-Salmonella* agar showed pink colour colonies in *S*.*dysenteriae* inoculated plate (lower panel) and no colonies were found in the control plate (upper panel). B) Appearance of orange/pink colour colonies in *S*.*dysenteriae* inoculated plate (lower Panel) and no colonies were observed in the control plate (upper panel).

Clinical isolates of *S*.*dysenteriae* showed pink colour colonies in *Shigella-Salmonella* Agar as seen in Fig 1A (lower panel) which confirmed that the strain used was *Shigella*.

The virulent nature of the *S*.*dysenteriae* was analyzed by Congo-red binding assay, appearance of orange/pink colour colonies of *S*.*dysenteriae* (Fig 1B lower panel) suggest that these species are highly virulent when compared to control (Fig 1B upper panel)

### Histological changes in rat ileal loop infected with *S*.*dysenteriae*

**Fig 2:**
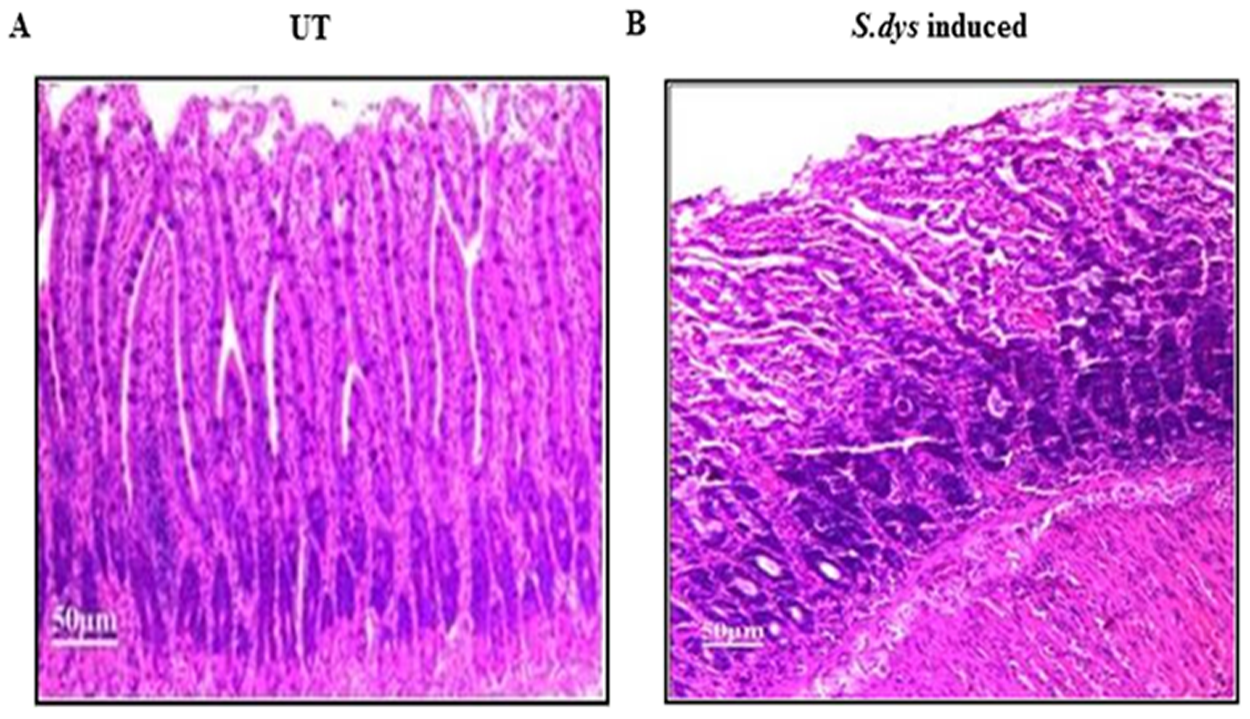
Histology of rat ileal loop infected with *S*.*dysenteriae*. A) Control rat ileal loop showed normal and elongated villi. B) *S*.*dysenteriae* infected rat ileal loop showed ulceration, inflammatory infiltration, broadening and congestion of the villi. Scale bar represents 50μM.

Histological examination revealed that control (UT) ileal loop of the rat showed normal architecture and elongation of the villi (Fig 2A). In contrast, *S*.*dysenteriae* infected rat ileal loops showed ulceration, inflammatory infiltration, broadening and congestion of the villi. Moreover, the villi architecture was altered during *S*.*dysenteriae* infection (Fig 2B).

### *S*.*dysenteriae* infection induces proinflammatory cytokines in rat ileal loop model

**Fig 3:**
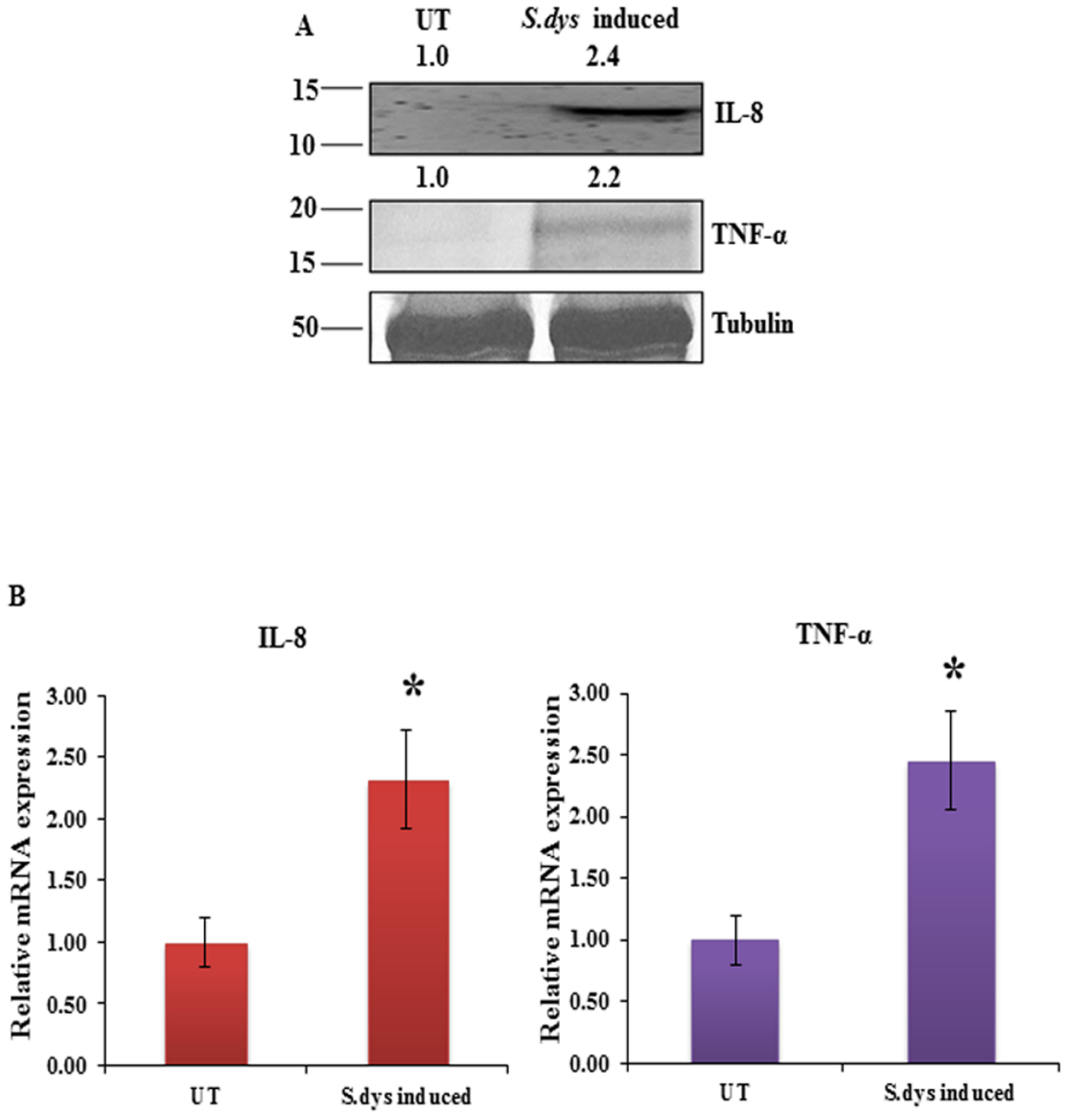
Activation of proinflammatory cytokines during *S*.*dysenteriae* infection. A) *S*.*dysenteriae* infected rat ileal loop protein lysates were analysed by western blots for the indicated proteins. B) IL-8 and TNF-α mRNA expression in *S*.*dysenteriae* infected rat ileal intestinal tissue lysate. IL-8 and TNF-α mRNA levels were normalized with GAPDH. Data are the mean ± SD. (n = 3). *, p <0.05.

To investigate whether *S*.*dysenteriae* mediated inflammation regulates proinflammatory cytokines in the *in vivo* rat ileal loop model, we assessed the expression of proinflammatory cytokines TNF-α and IL-8 in *S*.*dysenteriae* induced rat intestinal protein lysates. Our Immunoblot result revealed that IL-8 and TNF-α showed increased expression in *S*.*dysenteriae* induced rat intestinal protein lysate than in untreated (Fig 3A). Additionally, our real-time PCR data indicated that increased expression of IL-8 and TNF-α transcript level in *S*.*dysenteriae* induced rat intestinal tissue when compared to untreated (Fig 3B). The increase in cytokine profile demonstrates the evidence of tissue inflammation in *S*.*dysenteriae* induced rat ileal loop model.

### *S*.*dysenteriae* mediated inflammation alters localization of β-catenin and NF-κB in rat ligated ileal loop model

**Fig 4:**
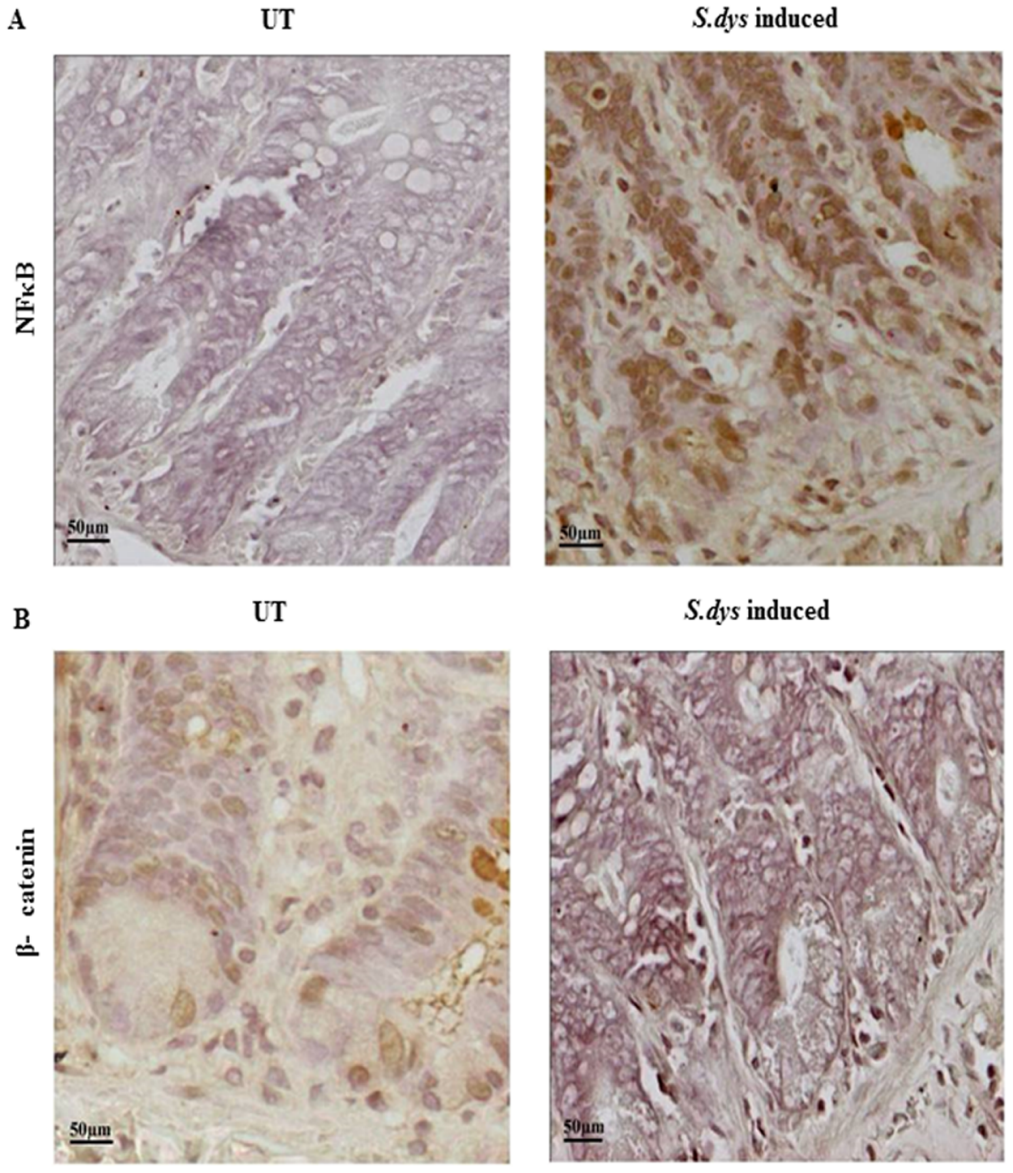
Differential expression of NF-κB and subcellular localization of β-catenin in rat ligated ileal loop model during *S*.*dysenteriae* infection. A) Mild expression of NF-κB was observed in lower part of the crypts in untreated tissue sections whereas increased expression of NF-κB was found in *S*.*dysenteriae* infected rat intestinal tissue sections by immunohistochemistry. Scale bar represents 50μM. B) Immunohistochemical analysis of β-catenin showed mild membranous expression in untreated sections whereas weak cytoplasmic expression was found in *S*.*dysenteriae* infected tissue sections. Scale bar represents 50μM.

Next we determined the mechanism by which *S*.*dysenteriae* infection regulates proinflammatory cytokines during inflammation. Since, NF-κB - β-catenin signaling pathway activates cytokines and chemokines in infected intestinal epithelial cells, we examined the subcellular distribution of β-catenin and NF-κB in our *in vivo* model. The immunohistochemistry results shown in Fig 4A (left panel) indicates that mild cytoplasmic expression level of NF-κB in untreated ileal section. In contrast, *S*.*dysenteriae* induced ileal sections (Fig 4A right panel) showed dramatic increase in NF-κB expression and interestingly it is translocated into the nucleus. The β-catenin staining in untreated ileal sections (Fig 4B left panel) were mostly located around the cell membrane and in the cytoplasm. In *S*.*dysenteriae* induced ileal sections we noted very weak β-catenin staining in the lower parts of the crypts (Fig 4B right panel).

### *S*.*dysenteriae* infection modulates β-catenin and NF-κB signaling pathways in rat ligated ileal loop model

**Fig 5:**
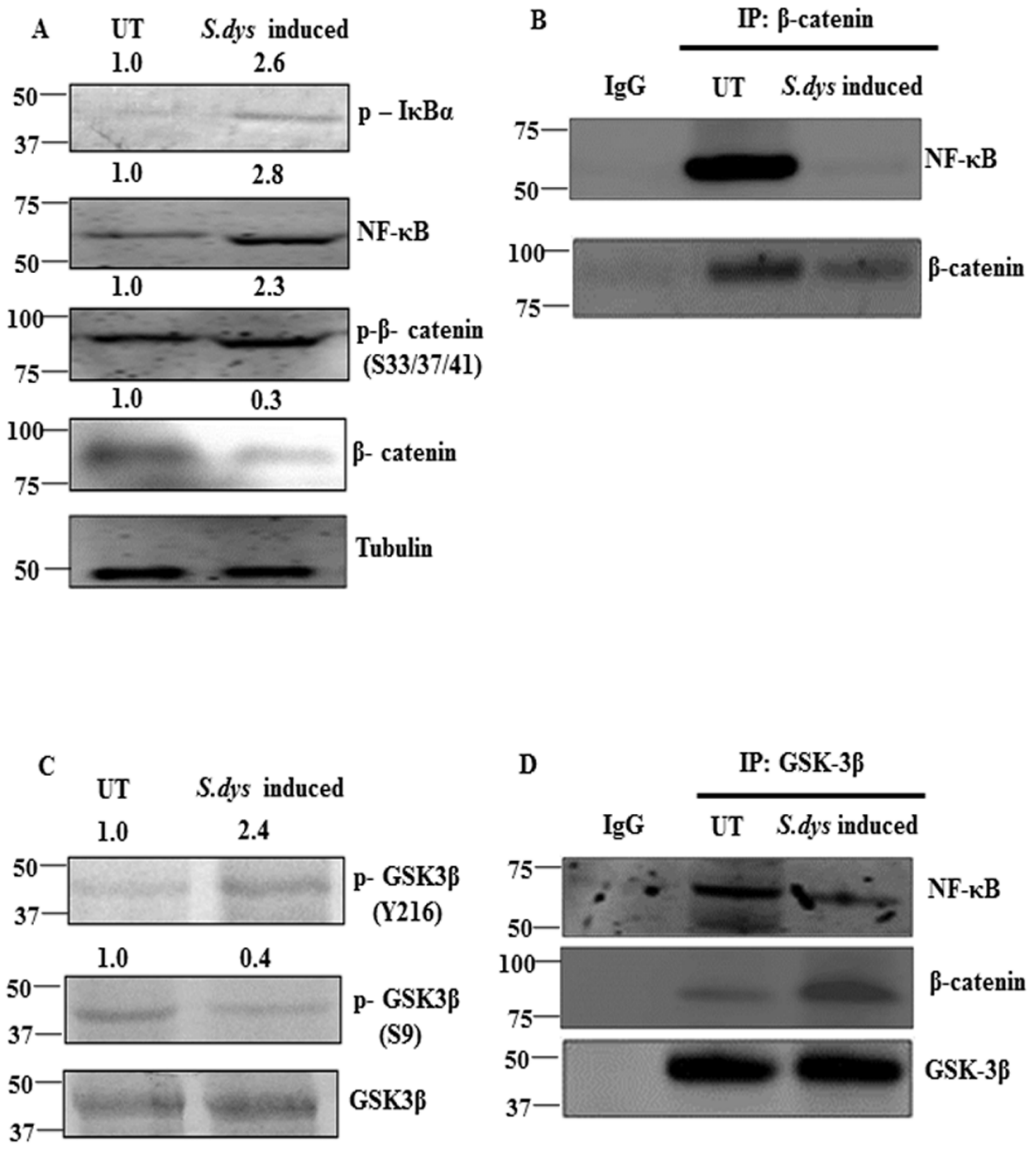
*S*.*dysenteriae* infection promotes binding of NF-κB and β-catenin through GSK-3β. A) *S*.*dysenteriae* infected rat ileal loop protein lysates were analysed by western blots for the indicated proteins. B) *S*.*dysenteriae* infected rat intestinal protein lysate were immunoprecipitated with β-catenin and immunoblotted with NF-κB and β-catenin. Nonspecific IgG was used as negative control for immunoprecipitation. C) *S*.*dysenteriae* infected rat ileal loop protein lysates analysed by western blots for the indicated proteins. D) Protein lysate from *S*.*dysenteriae* infected rat intestinal tissues were immunoprecipitated with GSK-3β and immunoblotted with NF-κB, β-catenin and GSK-3β. Nonspecific IgG was used as negative control for immunoprecipitation.

To determine the mechanism by which *S*.*dysenteriae* infection regulates differential expression of β-catenin and NF-κB in the ileal loop model, we analysed the expression of p-IκBα by western blot analysis which is an indicator of increased NF-κB activation. Our data demonstrated that *S*.*dysenteriae* infection increased p-IκBα expression when compared to untreated. We also noticed the increased expression of NF-κB in the *S*.*dysenteriae* infected tissue lysate which replicates our immunohistochemistry data (Fig 5A). To determine whether *S*.*dysenteriae* infection modulate β-catenin activity we assessed the expression of phospho- β-catenin by western blot analysis in our model. The subsequent degradation of β-catenin depends on the phosphorylation at S33 and S37 which is essential for β-catenin recognition by the ubiquitin ligase β-TrCP (17). As shown in Fig 5A *S*.*dysenteriae* infection increased the phosphorylation of β-catenin at S33 and S37 and reduced total β-catenin expression level when compare to untreated which indicates the degradation of β-catenin.

To investigate whether *S*.*dysenteriae* infection modulates interaction between NF-κB and β-catenin complex in the rat ileal loop model, we immunoprecipitated β-catenin and probed for NF-κB. The NF-κB/ β-catenin complex was dramatically reduced in *S*.*dysenteriae* induced rat intestinal protein lysate than the untreated (Fig 5B). Immunoprecipitation with IgG was used as a negative control and failed to pull down NF-κB. Taken together, this finding indicates that *S*.*dysenteriae* infection alters the interaction between NF-κB/ β-catenin complex in our *in vivo* model.

GSK-3β that acts as critical negative regulator of β-catenin also phosphorylates IκB, the inhibitor of NF-κB activity. As GSK-3β play a dual activity in the interaction between NF-κB and β-catenin pathways, we evaluated the activity of GSK-3β in the *S*.*dysenteriae* induced rat ileal loop model. Specific phosphorylation of GSK-3β at Ser9 leads to inactivation of its kinase activity whereas at Tyr216 stimulates GSK-3β kinase activity resulting in phosphorylation and degradation of β-catenin (19). In our ileal loop model *S*.*dysenteriae* infection increased the phosphorylation of GSK-3β Tyr216 (Fig 5C) and in contrast decreased phosphorylation of GSK-3β at (Ser9) (Fig 5C) which leads to β-catenin degradation and supports our Fig 5A data where the total amount of β-catenin decreased. *S*.*dysenteriae* infection does not alter the total amount of GSK-3β (Fig 5C). These data indicates that *S*.*dysenteriae* infection regulates β-catenin pathway by modulating GSK-3β activity.

Further to examine the role of GSK-3β on *S*.*dysenteriae* induced NF-κB activity we evaluated the interaction between GSK-3β /NF-κB/ β-catenin complex. Immunoprecipitation with GSK-3β was able to pull down more β-catenin in *S*.*dysenteriae* infected lysate where as GSK-3β /NF-κB complex was found more in the untreated lysate. These data indicates that *S*.*dysenteriae* infection promotes β-catenin degradation by forming GSK-3β /β-catenin complex and promotes NF-κB activity by dissociating interaction between GSK-3β /NF-κB (Fig 5D). Therefore, GSK-3β functions as a critical negative regulator of β-catenin.

## Discussion

**Fig 6:**
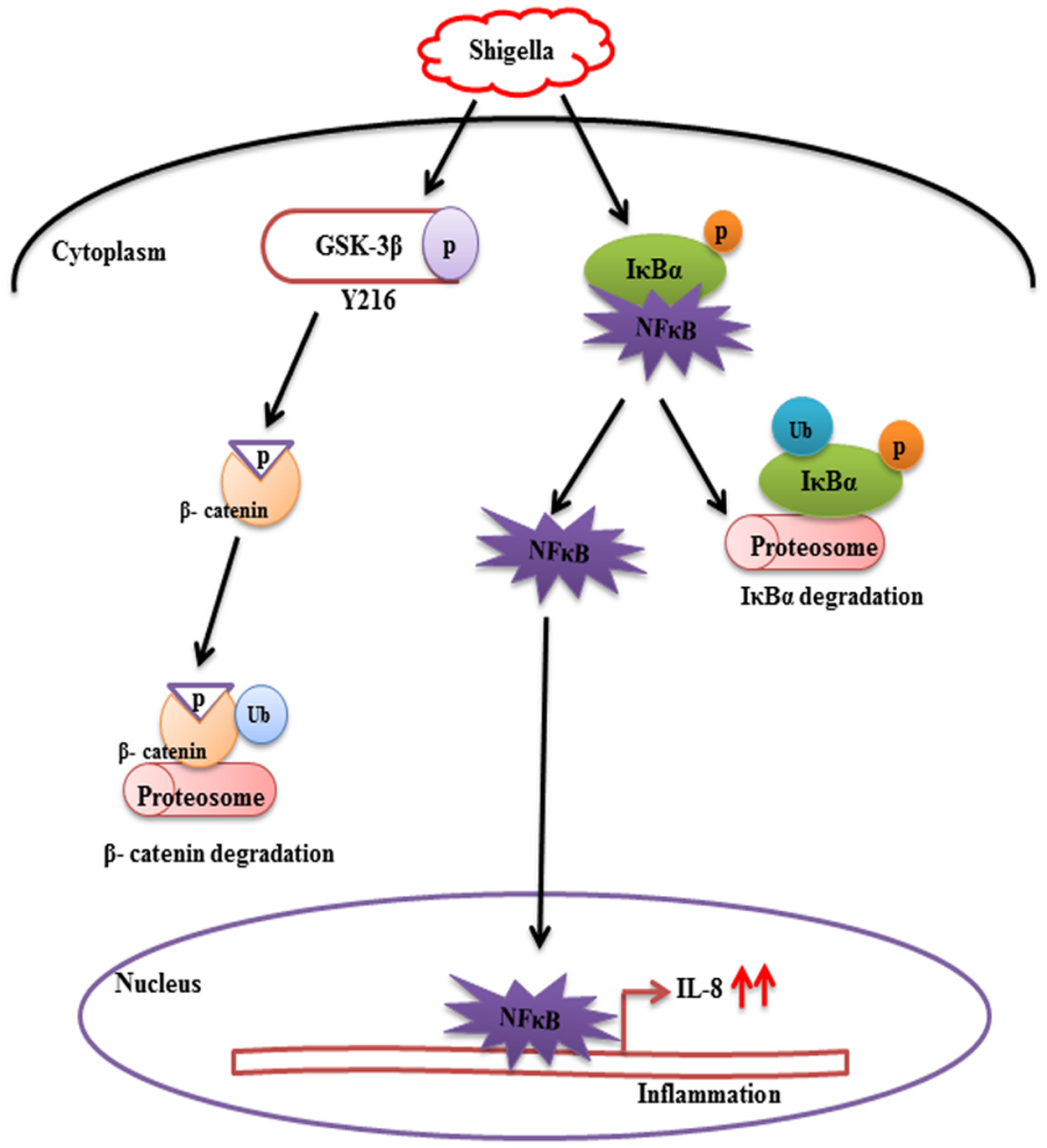
Proposed model depicting β-catenin/NF-κB role in regulating *Shigella*-induced proinflammatory cascade. *S*.*dysenteriae* stimulated phosphorylation of β-catenin through GSK-3β and β-catenin is subsequently degraded in the proteosome, simultaneously NF- κB is liberated from IκBα and it is degraded in the proteosome as that of β-catenin and NF- κB freely translocate to the nucleus. NF- κB activates transcription of IL-8 and other proinflammatory genes in the nucleus, thus leading to inflammation.

This study determines how *S*.*dysenteriae* induced proinflammatory cytokines is being modulated through the signaling mechanism which plays a major role in the regulation of intestinal inflammation. Our results demonstrate that β-catenin negatively regulates *Shigella* induced NF- κB activity which in turn increases IL-8 secretion (Fig 6).

Reports state that when cells are stimulated with Wnt proteins or pathogenic bacteria, the function of Wnt/β-catenin to act as the anti or pro-inflammatory role may depend on the stimulus, cell type and its crosstalk with other signaling pathways [29]. In the present study we identified that β-catenin act as the critical regulator of the inflammation in *S*.*dysenteriae* induced rat ileal model. Previous reports state the pro-inflammatory role of Wnt/*β*-catenin in 3T3-L1 preadipocytes stimulated with Wnt1 and the expression of the proinflammatory cytokines interleukins (IL)-6, IL- 12, and IFN*γ* upon activation of Wnt/*β*-catenin pathway by Wnt3a in mouse microglial cells [30,31]. Our results are also in accordance with previous reports that β-catenin acts as a negative regulator of inflammation during *Salmonella* infection in both *in vivo* and *in vitro* model [17]. In contrast there are also reports showing an anti-inflammatory role of Wnt/*β*-catenin pathway in mouse colon epithelial stem cells and macrophages infected with *Salmonella* [32] or *Mycobacterium* [33] which indicates that proinflammatory responses are downregulated in certain bacterial infections upon activation of Wnt/*β*-catenin pathway [19,34]. Here, we demonstrated that β-catenin acts as a negative regulator of inflammation in *S*.*dysenteriae* induced rat ileal loop model.

Previous report state that in response to pathogenic bacteria, β-catenin is phosphorylated by GSK-3β and as a result the physical interaction between β-catenin and NF-κB is inhibited. Phosphorylated β-catenin is subsequently degraded, liberating NF-κB from its physical connection i.e. IκBα the negative regulator of NF-κB which is degraded in a similar manner as that of p- β-catenin and as a result NF- κB enters into the nucleus and increases the secretion of proinflammatory cytokines like IL-6, IL-8, TNF-α that leads to inflammation [17]. We also showed increased p-β-catenin expression in *S*.*dysenteriae* infected rat ileal loop model.

Report on Toll-like receptor (TLR) agonist-stimulated monocytes showed that after TLR activation, GSK-3β plays a crucial role in controlling pro or anti-inflammatory response [35] and that the inhibition of GSK-3β with LiCl alters NF-κB activity through cross-regulation with β-catenin in colon and breast cancer cells [22]. The phosphorylation of GSK-3β and β-catenin in regulating inflammatory cascade is not only unique to *Salmonella* infection however there are also reports regarding other Type three secretory system (TTSS) dependent pathogens involved in similar mechanism as described earlier. Reports state that Yersinia stimulates GSK-3β phosphorylation during the early stages of macrophage infection [36] and Ipa effector proteins of TTSS play important role in *Shigella* induced infections and it is shown that IpaC associated β-catenin is tyrosine phosphorylated and destabilized, thus allowing bacterial invasion [37]. Previous report state that specific phosphorylation of GSK-3β at Tyr216 stimulates GSK-3β kinase activity resulting in phosphorylation and degradation of β-catenin [19]. Moreover, our data clearly showed increased GSK-3β phosphorylation at Tyr216 in *S*.*dysenteriae* infected rat ileal model which clearly indicates that β-catenin degradation is exclusively dependent on GSK-3β phosphorylation site. In accordance with these previous reports and from our data it clearly indicates that GSK-3β promotes degradation of β-catenin through ubiquitination, which prevents nuclear localization of β-catenin and activates the target gene IL-8 that leads to inflammation. Previous studies from our laboratory clearly showed that NF-κB is activated by TLR4 pathway leading to upregulation of IL-1β secretion during *S*.*dysenteriae* infection [38]. Therefore our data suggest that increased GSK-3β dependent phosphorylation of β-catenin and upregulation of IL-8 in *S*.*dysenteriae* infected rat ileum could be via TLR4 dependent NF-κB activation.

Our results of this study provide new insights into how proinflammatory cytokines are upregulated by β-catenin-NF-κB pathway during *S*.*dysenteriae* infection in rat ileal loop model. However, further studies are required to elucidate the mechanism that regulates crosstalk between Wnt/β-catenin and NF-κB pathways and crypt cell maintenance in the intestinal epithelial cells infected with *S*.*dysenteriae*.

## Supporting information

S1 Checklist “NC3Rs ARRIVE guidelines checklist. (PDF)
